# Lung compartmentalization of inflammatory biomarkers in COVID-19-related ARDS

**DOI:** 10.1186/s13054-021-03513-9

**Published:** 2021-03-24

**Authors:** Youenn Jouan, Thomas Baranek, Mustapha Si-Tahar, Christophe Paget, Antoine Guillon

**Affiliations:** 1 INSERM, Centre d’Etude des Pathologies Respiratoires (CEPR), UMR 1100, Tours, France; 2 Faculté de Médecine de Tours, Université de Tours, Tours, France; 3 Service de Médecine Intensive Réanimation, Centre Hospitalier Régional Universitaire, 2 Bd Tonnellé, 37044 Tours Cedex 9, France; 4 Service de chirurgie cardiaque et de réanimation chirurgicale cardio-vasculaire, Centre Hospitalier Régional Universitaire, Tours, France

## Dear Editor,

We read with great interest the article of Bendib et al*.* published in Critical Care [1], in which they systematically assessed inflammatory mediators in pneumonia-related ARDS, both in airways and blood compartments. The authors observed a lung compartmentalization of inflammatory mediators, with important heterogeneity in the bronchoalveolar lavage fluid-to-serum concentration ratios across the mediators screened. The concept of compartmentalization of inflammation has already been formulated in pneumonia [2], and issues raised by assessing lung inflammation using blood inflammatory markers has also been highlighted, as well as the subsequent limitations of these biomarkers for bedside management [3]. However, we believe that it is critical to examine it further in ARDS, in this COVID-19 era. Indeed, since the beginning of the pandemic, most of the studies exploring immune dysregulation during COVID-19 were nevertheless based on data obtained only from blood, not because this is the most relevant compartment, but because it is the most easily accessible.

**Fig. 1:**
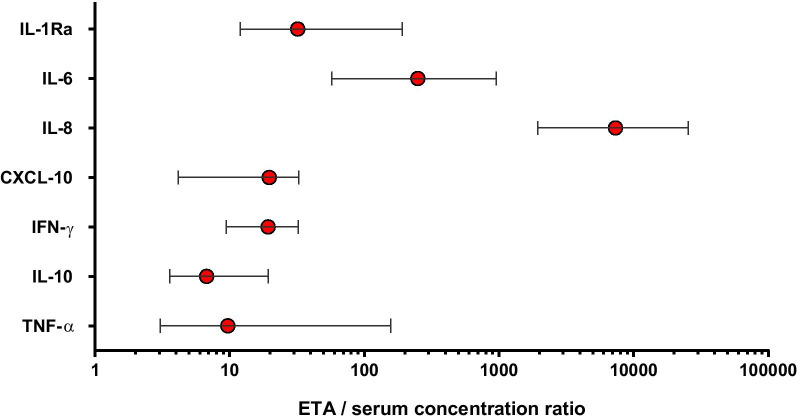
Endotracheal aspiration (ETA)-to-serum concentration ratio of inflammatory biomarkers in COVID-19-related ARDS. Inflammatory mediators (IL-1Ra, IL-6, IL-8, IL-10, CXCL-10, IFN-γ, TNF-α) were measured in blood (serum) and supernatants of endotracheal aspirations with the Bio-Plex Pro Human cytokines panel (Bio-Rad) in a multiplex fluorescent bead assay (Luminex), according to the manufacturer’s instructions. The density ratio of endotracheal aspiration (g/mL) was 1:1. Circles and bars represent median values and interquartile range respectively

In complement to the work of Bendib et al., we evaluated coincident inflammatory mediators in blood and respiratory fluids (endotracheal aspirates [ETA]) of 21 critically ill COVID-19 patients with ARDS requiring mechanical ventilation, within 48 h of their admission in ICU. As observed by Bendib et al., we found an increased ETA-to-blood concentration ratio for IL-8, the cytokine for which concentration was the most compartmentalized to the lung. However, in our COVID-19 ARDS cohort, the median (quartile 1; quartile 3) ratio was highly elevated: 7355 (1959; 22433), compared to 20 in the study of Bendib et al. Moreover, ETA to blood concentration ratios of IL-1RA, IL-6, IFN-γ, TNF-α and CXL10 were also highly elevated (Fig. 1), at a higher level than those reported by Bendib et al. for ARDS without shock. Thus, during COVID-19-driven ARDS -and even compared to non-COVID-pneumonia-related ARDS- inflammation appears highly compartmentalized to the lungs. These results are in line with publications reporting relatively low levels of systemic inflammatory mediators compared to other conditions requiring ICU [4, 5]. Taken together, these data challenge the concept of “systemic cytokine-storm” that has been employed to describe immune dysregulation during severe COVID-19. Consequently, in our quest of identifying reliable biomarker in pneumonia-induced ARDS -whether COVID or not-, lungs should not be excluded.

## Data Availability

The datasets used and/or analyzed for this research letter are available from the corresponding author on reasonable request.
