# Supplementary material for: Lung compartmentalization of inflammatory biomarkers in COVID-19-related ARDS
Source: Crit Care. 2021 Mar 24;25:120. doi: 10.1186/s13054-021-03513-9 (PMC7988241; doi:10.1186/s13054-021-03513-9)
Supplement: Related texts

# Reply: Authors’ response

**Authors:** Inès Bendib, Nicolas de Prost

**Affiliations:** 5 grid.50550.35 0000 0001 2175 4109 Service de Médecine Intensive Réanimation, Hôpitaux Universitaires Henri Mondor, Assistance Publique-Hôpitaux de Paris, 51, Avenue du Maréchal de Lattre de Tassigny, 94010 Créteil Cedex, France; 6 grid.410511.0 0000 0001 2149 7878 Groupe de Recherche Clinique CARMAS, Faculté de Santé de Créteil, Université Paris Est Créteil, 94010 Créteil Cedex, France

## Interleukin-8 is highly compartmentalized to the lungs in COVID-19 and non-COVID-19 ARDS

We thank Jouan et al. for their letter related to our recently published article [1]. Although serum biomarkers can depict specific profiles in patients with ARDS associated with COVID-19 and other diseases [6], the degree of cytokine release is markedly lower in critically ill COVID-19 than in other disorders associated with elevated cytokines (e.g., non-COVID-19 ARDS, sepsis, CAR-T cell therapy) [7]. Assessing the production of lung borne cytokines might thus be particularly relevant in COVID-19 patients. Consistent with our results in non-COVID-19 ARDS patients, Jouan et al. showed in patients with severe SARS-CoV-2 infection that the highest endotracheal aspirates (ETA) to blood concentration ratio was observed for interleukin-8 (IL-8). The authors pointed out some differences regarding the magnitude of the concentration gradient they measured (median value of the ETA to blood ratio of IL-8: 7355) and ours, which included only non-COVID-19 patients with pneumonia-associated ARDS (median value of the broncho-alveolar lavage (BAL) to serum ratio of IL-8: 21). Such magnitude difference might be due to the following factors: (1) a dilution of BAL fluid samples; (2) differences in IL-8 concentrations in proximal vs. distal airways and hypothetically, (3) higher IL-8 lung to blood concentration ratios in COVID-19 than in non-COVID-19 patients. ETA to blood concentration ratios of other cytokines, including interleukin-6 were also highly elevated in COVID-19 patients in the letter of Jouan et al. However, the clinical implications of these findings need to be investigated. Further studies will be needed to assess whether measuring lung to blood concentration ratios of selected biomarkers could help target patients most likely to benefit from immunomodulating drugs targeting cytokine pathways [8].
